# Thirty Minutes of Hypobaric Hypoxia Provokes Alterations of Immune Response, Haemostasis, and Metabolism Proteins in Human Serum

**DOI:** 10.3390/ijms18091882

**Published:** 2017-08-31

**Authors:** Jochen Hinkelbein, Stefanie Jansen, Ivan Iovino, Sylvia Kruse, Moritz Meyer, Fabrizio Cirillo, Hendrik Drinhaus, Andreas Hohn, Corinna Klein, Edoardo De Robertis, Dirk Beutner

**Affiliations:** 1 Department of Anaesthesiology and Intensive Care Medicine, University Hospital of Cologne, 50937 Cologne, Germany; Sylvia.kruse@uk-koeln.de (S.K.); hendrik.drinhaus@uk-koeln.de (H.D.); andreas.hohn@uk-koeln.de (A.H.); 2 Department of Otorhinolaryngology, Head and Neck Surgery, University of Cologne, 50937 Cologne, Germany; stefanie.jansen@uk-koeln.de (S.J.); moritz.meyer@uk-koeln.de (M.M.); dirk.beutner@uk-koeln.de (D.B.); 3 Department of Neurosciences, Reproductive and Odontostomatological Sciences, University of Naples “Federico II”, Via S. Pansini, 5-80131 Napoli, Italy; ivan4143@gmail.com (I.I.); fab.cirillo@gmail.com (F.C.); ederober@unina.it (E.D.R.); 4 CECAD Lipidomics & Proteomics Facilities, CECAD Research Center, University of Cologne, Joseph-Stelzmann-Str. 26, 50931 Cologne, Germany; corinna.klein@uni-koeln.de; 5 Department of Otorhinolaryngology, Head and Neck Surgery, University Medical Center Göttingen, Robert-Koch-Strasse 40, 37075 Göttingen, Germany

**Keywords:** pressure chamber, immune response, airline, hypobaric hypoxia

## Abstract

Hypobaric hypoxia (HH) during airline travel induces several (patho-) physiological reactions in the human body. Whereas severe hypoxia is investigated thoroughly, very little is known about effects of moderate or short-term hypoxia, e.g. during airline flights. The aim of the present study was to analyse changes in serum protein expression and activation of signalling cascades in human volunteers staying for 30 min in a simulated altitude equivalent to airline travel. After approval of the local ethics committee, 10 participants were exposed to moderate hypoxia (simulation of 2400 m or 8000 ft for 30 min) in a hypobaric pressure chamber. Before and after hypobaric hypoxia, serum was drawn, centrifuged, and analysed by two-dimensional gel electrophoresis (2-DIGE) and matrix-assisted laser desorption/ionization followed by time-of-flight mass spectrometry (MALDI-TOF). Biological functions of regulated proteins were identified using functional network analysis (GeneMania^®^, STRING^®^, and Perseus^®^ software). In participants, oxygen saturation decreased from 98.1 ± 1.3% to 89.2 ± 1.8% during HH. Expression of 14 spots (i.e., 10 proteins: ALB, PGK1, APOE, GAPDH, C1QA, C1QB, CAT, CA1, F2, and CLU) was significantly altered. Bioinformatic analysis revealed an association of the altered proteins with the signalling cascades “regulation of haemostasis” (four proteins), “metabolism” (five proteins), and “leukocyte mediated immune response” (five proteins). Even though hypobaric hypoxia was short and moderate (comparable to an airliner flight), analysis of protein expression in human subjects revealed an association to immune response, protein metabolism, and haemostasis

## 1. Introduction

With an increase in altitude, atmospheric pressure and the partial pressure of oxygen (O_2_) decrease rapidly leading to decreased O_2_ availability [1]. Organisms at higher altitudes must adapt to the stress of limited oxygen availability relative to sea level to still be able to sustain aerobic metabolic processes [2]. This results in a condition termed as hypobaric hypoxia which stresses biological systems because of non-availability of steady uninterrupted supply of oxygen for mitochondrial metabolism [1]. Hypobaric hypoxia resulting from the lowered barometric pressure is unavoidable, un-modifiable and uniform for everyone at any given altitude [2]. The integrated response to hypoxic challenges in humans is poorly understood and a number of controversies exist regarding the timings of such adaptations, the degree of hypoxia in which they occur and the tissue specificity of alterations in gene expression and metabolism [2].

Hypobaric hypoxia causes complex changes in the expression of genes [3]. The reduced partial pressure of oxygen can trigger the onset of adaptive responses, aimed at increasing the local oxygen concentration by several complementary actions [4,5]. The cellular responses to hypobaric hypoxia are complex and characterised by alteration in the expression of a number of genes, including stress related genes and corresponding proteins that are necessary to maintain homeostasis [1].

Changes in specific gene expression levels as well as the protein levels are excellent indicators that the organism has mobilized metabolic pathways in response to a specific stimulus [1]. Genes and their products have a great potential to serve as indicators of hypoxic stress [6].

This condition of reduced tissue oxygen availability also causes a number of life-threatening clinical manifestations including shortness of breath, tachycardia, headache, dizziness, acute mountain sickness (AMS), high-altitude pulmonary edema (HAPE), and high-altitude cerebral edema (HACE), mental confusion and memory deficit, ophthalmological disturbances, cerebral haemorrhages, and sleep disturbances [7]. However, little is known on protein expression during and after short-term and moderate hypoxia.

For research on proteomes, various technologies have been available for several years. Those technologies are summarized under the term “proteomics” [8]. The advantage of a proteomic rather than a transcriptomics approach is that protein expression levels are measured directly, rather than being inferred from abundance of the corresponding mRNAs, which are imperfectly correlated to protein concentration because of variable rates of synthesis and differences in message stability [2]. The analysis of complex contexts failed for many years due to the large amount of generated data. Within the last years, computer-based systems have been introduced and possibilities changed [9].

Two-dimensional gel electrophoresis (2-DIGE) coupled with mass spectrometry (MS) for identification of proteins is a powerful approach to understand global protein dynamics in response to different stimuli [7]. The combination with bioinformatic network analyses is an integrative technology to analyse the change of innumerable proteins at a defined time [10,11]. Biological pathways can represent complex processes at molecular level and can be a valuable aid for computational and experimental research utilizing the Omics data [12].

In the case of proteomic trials, a distinction can be made between methods for the separation of the individual protein species and methods for the characterization and identification: protein separation, chromatography, electrophoresis, identification or characterization, mass spectrometry, and sequencing.

Currently, only very few investigators have applied these technologies to the study of humans at altitude and in hypoxia chambers, and only under a limited number of hypoxic conditions and durations, and with usually small sample sizes [2]. Hypobaric hypoxia during airline travel (2300 m or 8000 ft [13]) induces several (patho-) physiological reactions in the human body [3].

The aim of the presented study was to measure the influence of moderate, hypobaric hypoxia on protein expression in the serum of healthy volunteers. To our knowledge, this is the first study designed to examine the effect of hypobaric hypoxia in a pressure chamber on the human proteome.

## 2. Results

### 2.1. Atmospheric Profile

**Figure 1:**
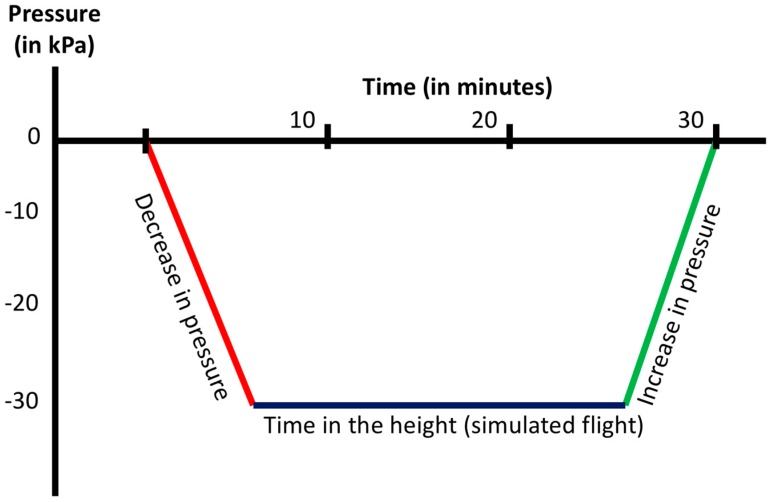
Pressure profile of the measurement: *X*-axis, time in min; *Y*-axis, pressure change in kPa. Phase 1 (red line): decompression phase; Phase 2 (blue line): isopression phase; Phase 3 (green line): compression phase.

During the first 1.5 min of the experiments, surrounding pressure was reduced by 0.24 bar (inside pressure 760 mbar) with a gradient of 0.2 bar/min. Following this, altitude was 2400 m (7874 ft) during the 30-min experiments. For the recompression phase, the same gradient was used (Figure 1).

### 2.2. Participants

Nine male and one female subjects (182 ± 7 cm and 85.2 ± 9.3 kg) with a mean age of 36.6 ± 5.9 years participated in the trial. Oxygen saturation of the subjects decreased from 98.1 ± 1.3% to a steady state of 89.2 ± 1.8% during 30 min of hypoxia (*p* < 0.001). None of the participants flew in an airplane within two weeks prior to this study.

### 2.3. Proteome Profiles in 2-DIGE

**Figure 2:**
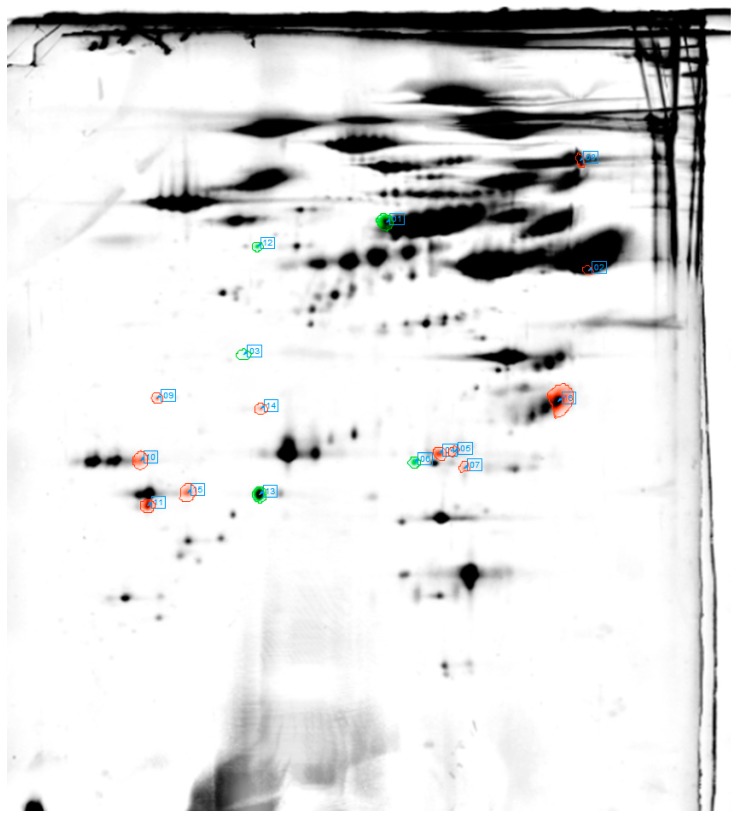
Proteins identified as significantly regulated two-dimensional gel electrophoresis (2-DIGE) and bioinformatic analysis. Red = upregulated; green = downregulated.

By analysing all protein spots on the 2-DIGE, expression of 16 protein spots was significantly altered. These spots were considered to be of interest and were, therefore, analysed more in detail. Of these, 14 were identified by matrix-assisted laser desorption/ionization followed by time-of-flight mass spectrometry (MALDI-TOF) representing 10 different proteins: Serum albumin (ALB; fold-change (F/C) −1.245), Phosphoglycerate kinase 1 (PGK1; F/C −1.203), Apolipoprotein E (APOE; F/C −1.294; +1.399; +1.419; +1.588), Glyceraldehyde-3-phosphate dehydrogenase (GAPDH; F/C +1.393), Complement C1q subcomponent subunit A (C1QA; F/C +2.109), Complement C1q subcomponent subunit B (C1QB; F/C +1.23; +1.38), Catalase (CAT; F/C −1.398), Carbonic anhydrase 1 (CA1; F/C −1.398), Prothrombin (F2; F/C +1.498), and Clusterin (CLU; F/C +1.267) (Table 1 and Figure 2).

### 2.4. Bioinformatic Analysis

**Figure 3:**
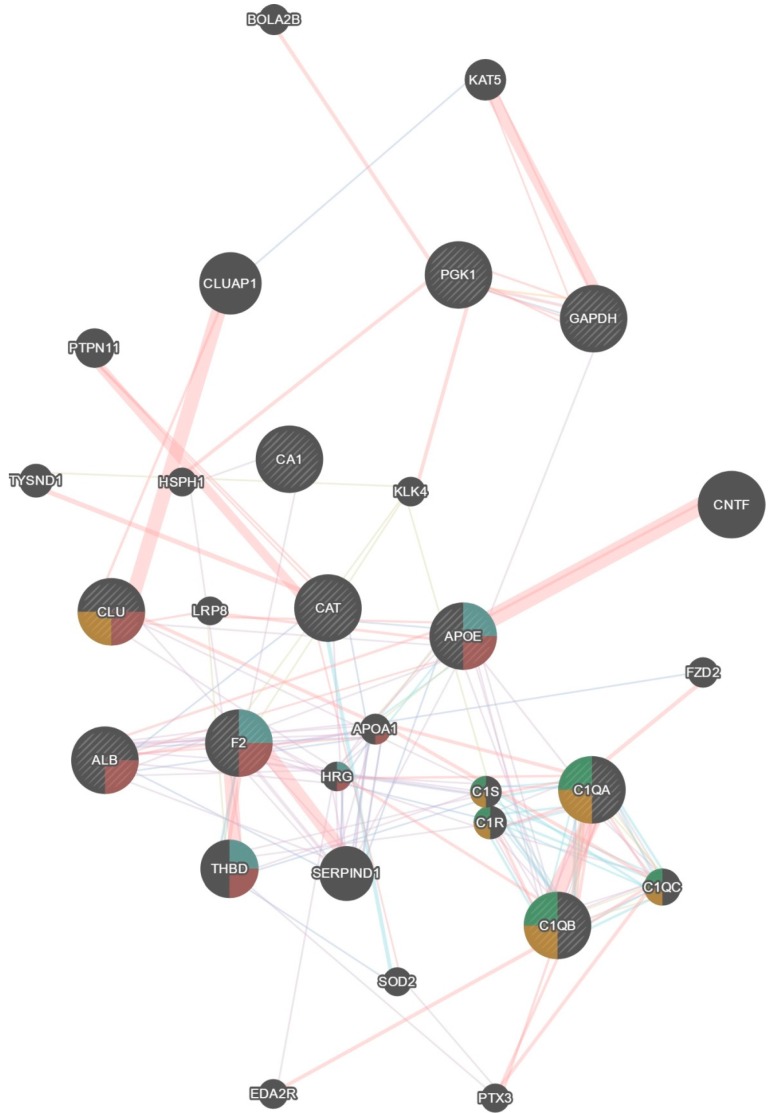
GeneMania^®^ network by the original dataset. Evidence shows four functions: regulation of haemostasis, platelet activation, humoral immune response, and B cell mediated immunity.

Bioinformatic analysis GeneMania software revealed an association of the altered proteins with the signalling cascades “regulation of haemostasis” (four proteins), “metabolism” (five proteins), and “leukocyte mediated immune response” (five proteins). Twenty additional proteins are involved in the network. Highlighting from this pool the functions with the greatest number of genes involved, an association of the altered proteins with signalling cascades of the following was found: (1) humoral response immunity (Clu, C1qa, C1qb, c1qc, c1s, and c1r); (2) platelet activation (F2, Apoe, Alb, Clu, apoa1, thbd, and hrg); (3) B cell mediated immunity (C1qa, C1qb, c1qc, c1s, and c1r); and (4) regulation of haemostasis (F2, Apoe, thbd, and hrg) (Figure 3).

### 2.5. Heat Map

**Figure 4:**
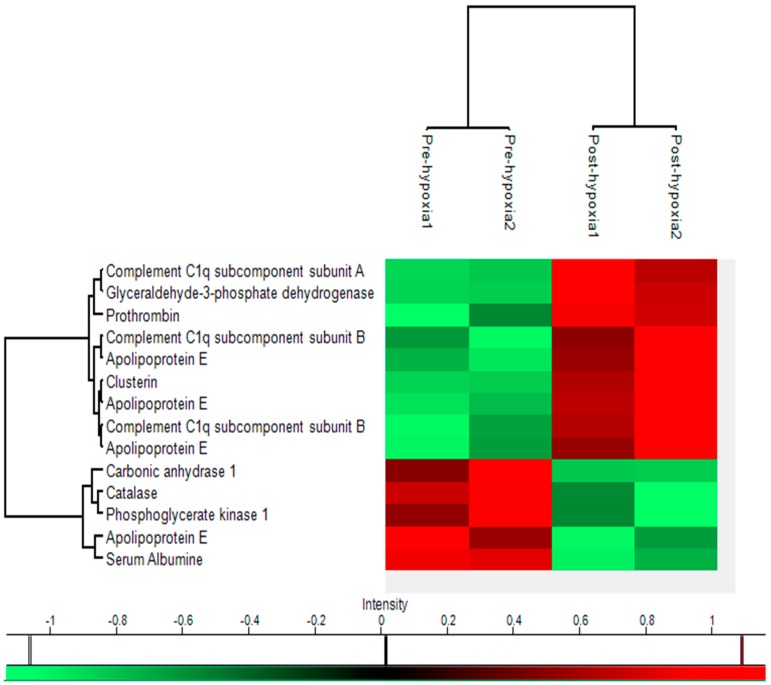
Categorical Cluster analysis (heat map) by Perseus^®^. The rows show the names of the proteins altered in the 12 spots. The columns show the timing groups of the experiment (before and after hypobaric hypoxia, each blood sample was obtained twice for each subject). An intensity colour scale to interpret the relative quantities from the heat map is shown.

Further bioinformatic analysis on quantitative data was performed with Perseus software. After converting raw values to a z-score, hierarchical cluster analysis was carried out and a heat map was generated (Figure 4). Two main clusters among the proteins were found and represent the group (group U: C1qa, C1qb, Gapdh, F2, Apoe, Clu) in which concentration was augmented after the procedure and the group (group D: Ca1, Cat, Pgk1, Apoe, Alb) in which the procedure brought about a reduction of concentration (Figure 4).

**Figure 5:**
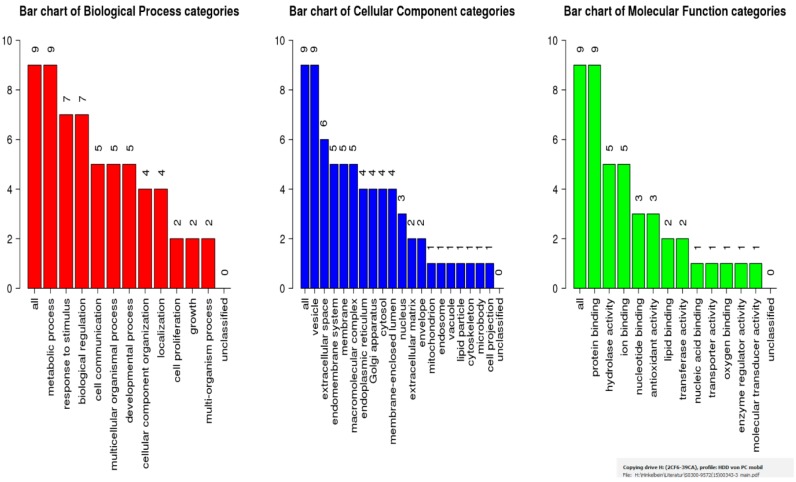
Categories of biological processes, cellular components, and molecular functions as identified by WebGestalt (Available online: http://genereg.ornl.gov/webgestalt/). The GO Slim summary is based upon the nine unique Entrez Gene IDs. Among the nine unique Entrez Gene IDs, nine IDs are annotated to the selected functional categories and also in the reference gene list, which are used for the enrichment analysis. Biological Process, Cellular Component and Molecular Function categories are represented by red, blue and green bars, respectively. The height of the bar represents the number of user list genes observed in the category.

Using Webgestalt, an Enrichment Analysis was performed (Figure 5). “Metabolic process” (nine proteins), “response to stimulus” (seven proteins), and “biological regulation” (seven proteins) were found to have the highest scores for biological processes. For cellular components, “vesicle” (nine proteins) and “extracellular space” (six proteins) were found to be involved. “Protein binding” (nine proteins) was the most probable molecular function category.

**Figure 6:**
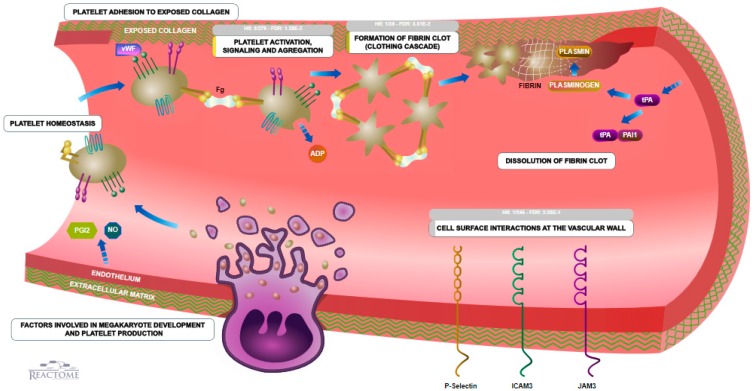
Haemostasis pathway fibrin clotting gathered by the website (available online: www.reactome.org). Platelet activation and formation of the fibrin clot are affected by the proteins found in the present study

KEGG analysis showed an involvement of two proteins (F2 and C1qrs) in the coagulation and complement cascades pathway, which was confirmed by reactome analysis (Figure 6).

## 3. Discussion

The aim of the presented study was to analyse changes in serum protein expression and activation of signalling cascades in human subjects staying 30 min in a simulated altitude equivalent to airline travel. Eight thousand feet (2400 m) was chosen as altitude since it is the typical pressure inside an aircraft cabin in-flight [13]. In this study, we used the proteomics technology to demonstrate the characteristic alterations of plasma proteins in moderate hypoxia after a simulated flight in a pressure chamber. The present study demonstrated significantly altered expression of 10 proteins after 30 min of a simulated airline flight. Analysis of serum proteins showed an association to immune response and haemostasis. All other proteins and protein spots on the 2-DIGE were statistically not different before and after the simulated flight.

### 3.1. Hypoxia Effects

With increase in altitude, atmospheric pressure and the partial pressure of oxygen decrease rapidly [3]. Oxygen availability also decreases resulting in situation termed as hypobaric hypoxia which stresses biological systems because of non-availability of steady uninterrupted supply of oxygen for mitochondrial metabolism [2].

The cellular responses to hypobaric hypoxia are complex and characterized by alterations in the expression of a number of genes, including stress related genes and corresponding proteins that are necessary to maintain homeostasis [14].

Chandel et al. could show that short-term reduction of O_2_ supply is followed by the modification of existing proteins through phosphorylation or other post translational changes [15]. Longer reduction of O_2_ can lead to cell death when the adaptive mechanisms are exhausted [16]. Hypoxia represents a major challenge for cells. That is why changes of gene expression, protein degradation and other post-translational modifications could be shown to result in alteration of the proteome [3].

### 3.2. Proteomics

The human proteome consists of approximately 20,000 gene products (proteins) [17,18]. Usually, there is a balance between the constant re-synthesis and simultaneous reduction of proteins [8]. Thus, the proteome is constantly undergoing changes in its composition, unlike the relatively static genome [9]. The regulation of these changes is complex and can be influenced by environmental stimuli, diseases and drugs. The proteome can, therefore, be described as the mirror of its environment and is highly dynamic.

The advantage of a proteomic approach, therefore, is that protein expression levels are measured directly. This could be shown to be more precise than measurement of corresponding mRNAs, which are imperfectly correlated to protein concentration due to variable rates of synthesis and differences in message stability [19].

The analysis of complex contexts failed for many years due to the large amount of generated data. Within the last years, computer-based systems have been introduced and analysability of generated data has been improved [9]. For exact analysis, several methods had to be used for many years. The combination of two-dimensional gel electrophoresis and mass spectrometry as well as bioinformatic network analysis is an integrative technology to analyse the change of innumerable proteins at a defined time [10,11].

In the case of proteomic trials, a distinction can be made between methods for the separation of the individual protein species and methods for the characterization and identification: protein separation, chromatography, electrophoresis, identification or characterization, mass spectrometry and sequencing.

Data analysis is one of the major challenges of modern MS-based shotgun proteomics experiments [20]. Using state-of-the-art technology, it has become possible to quantify several thousand proteins, and even complete proteomes within a single proteomics experiment [21,22].

### 3.3. Proteomics in High Altitude Research

Since its introduction in 1994 [23], proteomics has been rapidly developed as a research tool and widely applied to a vast array of science and medicine fields [24]. Application of the mass spectrometry (MS) technique to proteomic studies has improved accuracy and facilitated high-throughput screening, promoting the adoption of proteomics throughout life science research, as exemplified remarkably by biomarker screenings for disease diagnosis and physiological changes that accompany aging [24].

Proteomic studies have become an important part of high-altitude hypoxia analysis in recent years, and the findings have allowed for significant advances in our understanding of the mechanisms of hypoxia-related diseases and the corresponding treatments [24].

During the last decade, identification and quantitation of proteomes have been facilitated by the constant developments in mass spectrometry instrumentation, fractionation techniques, quantitation-strategies, and data analysis software [25]. In 2DE, proteins are separated according to their isoelectric points (pIs) and molecular weights (MWs), respectively, per dimension, forming a distribution pattern in gel (as protein spots) and providing visual information of the protein profile for the features of pH (through pIs), size (through MWs), and expression level (through spot intensity) [24]. The differentially expressed proteins (for example, when comparing the profiles of poor/non-adaptors vs. adaptors) are then selected, digested by trypsin, and subjected to MS for identification [24].

### 3.4. Hypoxia Protein Expression in the Present Study

In the present hypoxia study, 10 different proteins (CAT, CA1, APOE, ALB, PGK1, C1QA, C1QB, CLU GAPDH, and F2) were identified to be significantly regulated during the hypoxia phase. Catalase (CAT) is a serum protein involved in oxidative stress and antioxidant defences [26]. In the present study, expression of CAT was decreased after hypobaric hypoxia. Wadley et al. also found changes in the expression of CAT in the serum in cyclists at altitude but an up-regulation and not a down-regulation [27]. This is in congruency to the results of Krzeszowlak et al. who also found increased CAT activity after a nine-day exposure to high-altitude conditions [26]. However, the duration in hypoxic conditions in both studies was longer as in the present study which could explain different regulation of CAT.

Clusterin (apolipoprotein J) is a 75–80 kDa disulphide-linked heterodimeric protein associated with the clearance of cellular debris and apoptosis [28]. It is encoded by the *CLU* gene on chromosome8 [29] and a molecular chaperone responsible for aiding protein folding. Therefore, CLU is involved in many diseases related to oxidative stress, including neurodegenerative diseases, cancers, inflammatory diseases, and aging [30,31,32]. In addition, CLU is involved in the Hypoxia inducible factor-1α (HIF-1α) pathway. HIF-1α directly regulates nuclear clusterin transcription by interacting with hypoxia response elements in the clusterin promoter [33]. Many of the hypoxic adaptations could be shown to be driven by HIF-1α which is a central regulator of the hypoxic response [34]. This was identified in cultured cells and animal models originally by its binding to a hypoxia response element in the human erythropoietin gene [35,36]. Hypoxia response elements containing HIF-1α binding sites were identified in genes encoding transferrin [37], vascular endothelium growth factor [38], inducible nitric oxide synthase, glucose transporter 1 (GLUT 1) [39], and several glycolytic enzymes, all playing important roles in systemic, tissues, or intracellular O_2_ homeostasis allowing for increased anaerobic ATP synthesis [3].

Another hallmark of cellular responses to hypoxia is the up-regulation of oxygen-independent metabolic pathways to supply the additional energy necessary for cell survival under diminished oxygen availability [40]. Thus, in hypoxia, enzymes involved in the glycolytic pathway and glucose transporters are upregulated by HIF-1α, including PGK 1. PGK1 was also found in the present study but it was down-regulated.

In the present study, APOE was found to be up-regulated in three spots, which could explain an association of APOE with hypobaric hypoxia. APOE was also found by Ahmad Y et al. [1] associated with hypoxia. In their study, it was significantly decreased 12 and 24 h after hypobaric hypoxia in rats (IF 0.76 vs. 0.52, respectively) [1]. Zhou et al. also found elevated levels of APOE after hypoxia and postulated that it protects brain cells from hypoxia and glutamate-induced apoptosis [41]. Although this is an interesting postulate that could also explain the increased expression in the present study, it was not possible to proof this hypothesis with the methods of our study. Ahmad et al. have investigated long-term effects after hypoxia in high altitude natives [2]. Congruently, apolipoproteins (APOA1) and complement proteins (C3 and C4A) have also shown to be altered in their expression in this study [2]. However, specifically these proteins were not significantly altered in the present study.

Expression of GAPDH was significantly increased in the present study (fold-change, +1.393). This finding is in agreement with the study of Sharma et al. who also found GAPDH being increased in rats after short-term hypoxia using proteomic techniques [42]. This finding is not completely new since Arnaud et al. already found an increased activity of GAPDH in the blood of high-altitude natives in the year 1979 [43].

### 3.5. Coagulation

Hypoxia is known to be associated with deep vein thrombosis [44] and venous thromboembolism [45]. The link between long-haul air travel and venous thromboembolism is a subject of continuing debate [46]. It remains unclear whether the reduced cabin pressure and oxygen tension in the airplane cabin create an increased risk compared with seated immobility at ground level [46]. Interestingly, prothrombin expression was increased after hypobaric hypoxia in our study subjects. This might be one of the multiple factors contributing to an increased incidence of thromboembolic diseases such as thrombosis, pulmonary embolism or stroke after air travel, colloquially termed “economy class syndrome”. In accordance to our study, Ninivaggi et al. also found in 15 healthy individuals an endogenous thrombin potential and demonstrated that hypoxia causes a prothrombotic state [45]. Although AJ Schreijer et al. analysed healthy volunteers during long haul (8 h) air travel, they also found compromised coagulations proteins in the blood [47]. In congruency to the present study, they also concluded that hypoxia, triggering systemic inflammation and platelet activation, leads to coagulation induction and degranulation of platelets. However, we have found this evidence even after short haul (30 min) of simulated flight in the serum of our volunteers.

Analysing proteins in the context of air travel and coagulation is not completely new. However, there are other results published, not finding an increased risk after hypoxia. As Toff et al. and others have shown in their studies, the findings did not support the hypothesis that hypobaric hypoxia, of the degree that might be encountered during long-haul air travel, was associated with prothrombotic alterations in the haemostatic system in healthy individuals at low risk of venous thromboembolism [46,48,49]. Taken together, results of the present study cannot prove that hypoxia increases the risk of thromboembolic events, but do show—at least—an altered expression of proteins involved in coagulation.

### 3.6. Biological Pathways

Recent advances in proteomic techniques make it possible to monitor plasma protein expression profiles providing a better insight into the mechanisms involved in functional adaptations of cells, tissues, organs, and the whole organism in the hypoxic environment [2]. Hypobaric hypoxia can stress the biological system because of non-availability of oxygen supply for mitochondrial metabolism. Hypobaric hypoxia limits the availability of oxygen for reduction to H_2_O at cytochrome oxidase [7].

Paul et al. analysed 154 differentially expressed proteins upon HH exposure using Ingenuity Pathway Analysis (IPA) tool, without the constraint of using a single organism or tissue type, to determine the most significant pathways and networks that are perturbed across a range of HH conditions [50]. This is a very interesting approach since it describes many altered pathways in hypobaric hypoxia. The authors also found acute phase signalling as well as mitochondrial dysfunction pathways being affected which is in congruency to the present study. In addition, Liao et al. performed an interesting study with an innovative approach to identify hypoxia-induced alterations in the serum of volunteers at 5300 m [51]. They found that hypobaric hypoxia caused significant and comprehensive metabolic changes, as represented by significant changes of 44 metabolites and four relevant enzymes. Using MetaboAnalyst 3.0, it was found that several key metabolic pathways were acutely perturbed [52].

Besides these results, Tyagi et al. provide some other explanations for hypoxia-induced alterations of the coagulation [52]. In their study, the authors investigated the role of platelet proteome/reactivity to elucidate the acute hypoxia-induced prothrombotic reaction. Proteomic analysis of hypoxic platelets revealed 27 differentially expressed proteins, including those involved in coagulation [52]. Interestingly, patients who developed thrombosis while at extreme altitude had elevated plasma calpain activities and increased soluble P-selectin level. In the light of the present study, it may not only hypoxia and alterations in the serum proteins of humans but also changes in the proteome of platelets which can result in an acute hypoxia-induced prothrombotic reaction.

### 3.7. Limitations

Limitations of the presented study clearly are that there was no control group. Ten participants were included in the study and measured in the pressure chamber with a simulated flight of 30 min. Obviously, most flights take more time. It is very interesting, anyways, that even this short hypoxic time period seems to lead to a reaction in the immune response and haemostasis. Furthermore, our study population was mainly male. Therefore, results should be interpreted carefully.

## 4. Materials and Methods

### 4.1. Inclusion and Exclusion Criteria

The Ethics Committee of the University Hospital of Cologne, Germany, approved this trial (No. 15-025, 29 January 2016, Ethics Committee, University Hospital of Cologne) and participants signed a written consent for the study. Participants were healthy adults and had never had ear surgery or problems with pressure equalization before. Other exclusion criteria were pregnancy and claustrophobia. The trial was registered prior to all study-related interventions in the German Clinical Trials Register (No. DRKS00009023; Available online: http://apps.who.int/trialsearch/).

### 4.2. Simulated Flight in the Pressure Chamber

For the simulation of each flight, a hypo-/hyperbaric pressure chamber (Haux Life Support, Karlsbad, Germany) was used to create a situation of hypobaric hypoxia. This pressure chamber is used for experimental purposes only [53,54,55]. In the pressure chamber, the environmental pressure and oxygenation can be changed. Due to these altered environmental conditions, changes in protein expression (proteome) are also likely.

Although problems after long-haul flights are common and were investigated before, there is little known about short-haul flights. Therefore, duration of moderate hypoxia (simulation of 2400 m) was 30 min in the hypobaric chamber (2 subjects per run) (Figure 1).

Participants were placed in the pressure chamber and the door was closed. Take-off of an aircraft was simulated from normal outside air pressure. The pressure change rate during decompression was 0.2 bar/min (decompression phase). After 1.5 min, surrounding pressure was reduced by 0.24 bar, equivalent to 2400 meters height (inside pressure 760 mbar). Subjects were left there for 30 min (isopression phase). After this time, chamber pressure increased again until ambient pressure was reached again. During the time in the pressure chamber, blood oxygenation was documented for each participant using pulse oximetry. Fresh air was supplied during these 30 min to secure a stable O_2_ concentration inside the pressure chamber. 

### 4.3. Collection and Isolation of Serum Samples

Before and after hypobaric hypoxia in the hypobaric chamber, serum was drawn and centrifuged at 5000× *g* for 5 min for further analysis. Serum samples were stored at −80 °C until analysis. All samples used in this study were prepared within 30 min of sample collection and showed no signs of haemolysis.

### 4.4. Depletion of High-Abundant Proteins

For depletion of high-abundant proteins from human serum, the Agilent Multiple Affinity Removal System (Agilent, 5301 Stevens Creek Blvd, Santa Clara, CA, USA) was used. This system removes the 14 high abundant proteins via polyclonal antibodies packed into an HPLC column. Samples processing was performed according to the manufactures protocol.

### 4.5. Sample Preparation for 2DE Electrophoresis

The pooled serum (5 mg of 10 sera before and after hypoxia) was diluted with four volumes of buffer A (Agilent Multiple Affinity Removal Buffer A, Agilent, Waldbronn, Germany) and spun through a 0.22 mm spin tube at 16,000× *g* at room temperature for 2 min. A 200 µL sample was injected onto a 4.6 × 50 mm^2^ affinity column in buffer A at a low rate of 0.25 mL/min for 10 min. The bound fraction was eluted with buffer B at a flow rate of 1.0 mL (min for 3.5 min). Then, the column was re-equilibrated with buffer A for 10 min.

Flow-through was collected at 1.5–4.5 min automatically into deep well plates. After HPLC, the collected samples were precipitated with TCA. Then the pellets were solved in 9 M urea, 2% ampholytes, and 70 mM DTT. After incubation for 30 min and centrifugation for 45 min at 15,000× *g*, the supernatant was frozen in new tubes at −80 °C. The protein concentration was determined with Bradford assay.

### 4.6. Two-Dimensional Gel Electrophoresis (First and Second Dimension)

Two-dimensional gel electrophoresis (2-DEGE) was performed by Proteome Factory AG (Berlin, Germany) based on the protocol by Klose and Kobalz 1995 [56]. Two hundred micrograms of protein were applied to vertical rod gels (9 M urea, 4% acrylamide, 0.3% PDA, 5% glycerol, 0.06% TEMED, and 2% carrier ampholytes (pH 2–11), 0.02% APS) for isoelectric focusing at 8820 V·h in the first dimension. After focusing, the IEF gels were incubated in equilibration buffer, containing 125 mM triphosphate (pH 6.8), 40% glycerol, 65 mM DTT, and 3% SDS for 10 min and frozen at −20 °C.

Subsequently, second dimension SDS-PAGE (20 cm × 30 cm × 0.1 cm) were prepared containing 375 mM. Tris HCL buffer (pH 8.8), 12% acrylamide, 0.2% bisacrylamide, 0.1% SDS, and 0.03% TEMED. After thawing, the equilibrated IEF gels were immediately applied to SDS–PAGE gels. Electrophoresis was performed using 140 V for 5 h 15 min until the front reached the end of the gel.

### 4.7. Identification of Relevant Protein Spots

After 2DE separation, the gels were stained with FireRuth (Proteome Factory, PS-2002, Berlin, Germany). For identification of altered proteins due to hypoxia, gel-pairs (before vs. after hypoxia) were compared. Biomathematical spot identification was performed with Δ2D (Decodon, Greifswald, Germany). Spots were considered significant if the induction factors were >2 or <0.5 with a *p* value of less than 0.01. Significantly altered spots were identified on the gel and used for MALDI-TOF protein identification.

### 4.8. MS Identification of Proteins

Protein identification using nano LC-ESI-MS/MS was performed by Proteome Factory. The MS system consisted of an Agilent 1100 nanoLC system (Agilent, Waldbronn, Germany), PicoTip electrospray emitter (New Objective, Woburn, MA, USA) and an Orbitrap XL or LTQ-FT Ultra mass spectrometer (ThermoFisher, Bremen, Germany). 

Protein spots were in-gel digested by trypsin (Promega, Mannheim, Germany) and applied to nanoLC-ESI-MS/MS. Peptides were trapped and desalted on the enrichment column (Zorbax SB C18, 0.3mm × 5 mm, Agilent) for five min using 2.5% acetonitrile/0.5% formic acid as eluent, then peptides were separated on a Zorbax 300 SB C18, 75 µm × 150 mm column (Agilent) using an acetonitrile/0.1% formic acid gradient from 5% to 35% acetonitril within 40 min. MS/MS spectra were recorded data-dependently by the mass spectrometer according to manufacturer’s recommendations.

Proteins were identified using MS/MS ion search of the Mascot search engine (Matrix Science, London, UK) and swissprot/uniprot database (Available online: http://www.uniprot.org/). Ion charge in search parameters for ions from ESI-MS/MS data acquisition were set to “1+, 2+, or 3+” according to the instrument’s and method’s common charge state distribution.

### 4.9. Bioinformatic Analysis of Proteins

Significantly altered proteins were used for further bioinformatic analysis to identify underlying networks, signalling cascades, and pathways affected. Biological functions of regulated proteins were identified using functional network analysis.

### 4.10. Hierarchical Cluster Analysis

Heat maps are an efficient method of visualizing complex datasets organized as matrices [55]. Quantitative information, concerning only the proteins that altered significantly their expression, was gathered (two average values for each item and for each condition) and converted to TSV (Tab-Separated Values) text file using Microsoft Excel^®^ 2007. In this format the data were analysed using the free software Perseus (v. 1.5.8.5, Max Planck Institute of Biochemistry, Berlin, Germany), which performed the z-scoring and, consequently, the hierarchical cluster analysis. Perseus^®^ is a holistic software platform that allows continuous expansion of scalable analytical tools, their smooth integration and reusability while providing the user with explicit documentation of the analysis steps and parameters [57].

A heat map performs two actions on a matrix. First, it reorders the rows and columns so that rows (and columns) with similar profiles are closer to one another, causing these profiles to be more visible to the eye. Second, each entry in the data matrix is displayed as a colour, making it possible to view the patterns graphically [58].

### 4.11. Protein Interaction and Network Identification

GeneMANIA (Available online: http://www.genemania.org/) is a tool that helps to predict interactions and function of list of genes in form of network and, when available, of pathway [59,60]. GeneMANIA gives the possibility of customizing the network, allowing the choice of data sources or highlighting specific functions, with a more comfortable graphic experience [61]. It is developed and continually updated by the University of Toronto and is funded by the Ontario Ministry of Research and Innovation. GeneMANIA knowledge is based on data from large databases, which comprehend Gene Expression Omnibus, BioGRID, EMBL-EBI, Pfam, Ensembl, Mouse Genome Informatics, the National Center for Biotechnology Information, InParanoid, and Pathway Commons [14,15]. As these software programs use different algorithms, we chose to perform the bioinformatics analysis with all of them in order to retrieve the highest number of predicted interactions, maintaining an acceptable level of confidence (0.400).

### 4.12. Reactome

The Reactome Knowledgebase (Available online: http://www.reactome.org) is an open source tool for analysing and finding molecular details of signal transduction, transport, DNA replication, metabolism and other cellular processes of datasets provided by the user and permitting ID mapping, pathway assignment and over-representation or enrichment analysis [61]. Pathway annotations are cross-referenced to bioinformatics databases including NCBI Gene, Ensembl, and UniProt databases, the UCSC Genome Browser, the KEGG Compound and ChEBI small molecule databases, PubMed, and Gene Ontology. Reactome allows for visualization and exploration of the finished dataset as an interactive process map.

### 4.13. KEGG

KEGG (Available online: http://www.kegg.jp/ or http://www.genome.jp/kegg/) is an open source database resource of genes and genomes with the aim of assigning functional meanings to both at the molecular and higher levels. Molecular-level functions are stored in the KO (KEGG Orthology) database, where each KO is defined as a functional ortholog of genes and proteins (Available online: https://doi.org/10.1093/nar/gkw1092). Higher-level functions derived by datasets, especially large-scale datasets in genomics, transcriptomics, proteomics, and metabolomics are represented by networks of molecular interactions, reactions and relations in the forms of KEGG pathway maps, BRITE hierarchies and KEGG modules.

### 4.14. WebGestalt

WebGestalt (Available online: http://genereg.ornl.gov/webgestalt/) is a “WEB-based GEne SeT AnaLysis Toolkit” designed for functional genomic, proteomic and large-scale genetic studies (Available online: https://www.ncbi.nlm.nih.gov/pubmed/15980575?dopt=Abstract). It is composed by modules and allows users to upload gene sets, to compare them with different gene sets, to visualize various biological contexts, including Gene Ontology, tissue expression pattern, chromosome distribution, metabolic and signalling pathways, protein domain information and publications. It is possible to retrieve information for all genes in a gene set as well as perform statistical tests to suggest biological areas that are important to a gene set and warrant further investigation.

## 5. Conclusions

A broader understanding of hypoxia-induced alterations in cellular or organ function could be better achieved from a combined knowledge derived from the concerted application of genomic and proteomics approaches [1]. Even though hypobaric hypoxia was short and moderate (30 min at 2400 m; comparable to an airliner flight), analysis of serum protein expression in human subjects revealed an association to immune response and haemostasis. According to these results, even moderate hypobaric hypoxia seems to influence the human immune system. This may be a contributing factor to in-flight thrombosis (“economy class syndrome”) [60].

Using cells isolated from human subjects, such as oral swabs or blood buffy coat could be a future consideration for following studies. It is at least possible that the changes in serum proteins reflect endothelial or hepatic mild damage. Results of the present study may give input for developing molecular tests for identification of hypoxia-induced problems during airline flights.

The results in the presented study lead to further questions: What is the effect of the moderate hypoxia on the passenger in the aircraft directly? What do passengers need to know? Is there any danger? What happens after the aircraft has landed? It would also be worth investigating how long the observed changes in protein expression last after cessation of hypobaric hypoxia.

Future studies are needed with more participants and longer time of simulated flights to clarify these questions and should simulate flights in higher altitude to see even more effect on the proteome.

## Figures and Tables

**Table:** 

Original Dataset Proteins	GeneMania Predicted Proteins
Pgk1, Cat, Apoe, Clu, F2, Alb, C1qa, C1qb, Ca1, Gapdh	Bola2b, Eda2r, Ptx3, Sod2, Serpind1, C1qc, C1r, C1s, Thbd, Hrg, Apoa1, Fzd2, Lrp8, Cntf, Klk4, Hsph1, Tysnd1, Ptpn11, Cluap1, Kat5

**Table 1:** Proteins identified by matrix-assisted laser desorption/ionization followed by time-of-flight mass spectrometry (MALDI-TOF).

Name	Uniprot key	Gene	Function	Location	Pre-hypoxia Mean	Post-hypoxia Mean	*p*-value	Fold-change	Molecular Weight	*pI* value	Sequence Coverage	Mascot Score
Catalase	P04040	CAT	Protection from toxic effects of hydrogen peroxide, aid to cellular growth of e.g. T-cells, B-cells, AML cells, Melanome cells, etc	Cytosol, endoplasmatic reticulum, mitochondria, peroxisome	0.032	0.023	0.021	−1.398	59,719	6.9	63%	1941
Carbonic anhydrase 1	P00915	CA1	Bicarbonate transport, carbon metabolism	Cytosol, exosomes	0.416	0.298	0.038	−1.398	28,852	6.59	75%	1176
Apolipoprotein E	P02649	APOE	Cholesterol metabolism, Lipid metabolism, Lipid transport, Steroid metabolism, Sterol metabolism, Transport protein	Chylomicron, HDL, VLDL	0.056	0.043	0.029	−1.294	36,132	5.65	69%	1785
Serum Albumine	P02768	ALB	Binding capacity for water, Ca^2+^, Na^+^, K^+^, 0 fatty acids, hormones, bilirubin and drugs, regulation of the colloidal osmotic pressure of blood, zinc transporter	Serum, extracellular	0.474	0.381	0.007	−1.245	69,321	5.92	66%	2262
Phosphoglycerate kinase 1	P00558	PGK1	Gluconeogenesis, glycolysis, epithelial cell differentiation	Cytoplasm	0.021	0.017	0.033	−1.203	44,586	8.3	70%	1539
Complement C1q subcomponent subunit B	P02746	C1QB	Complement activation, innate Immune system, Inner ear development, Proteolysis	Blood microparticle, collagen, complement C1 complex, exosomes	0.157	0.193	0.022	1.23	26,704	8.83	39%	686
Clusterin	P10909	CLU	Apoptosis, complement system, immune defence, innate immune system	Cytoplasma, cytoplasmic vescicles, endoplasmatic reticulum, membrane, mitochondria, microsomes, nucleus	0.663	0.84	0.014	1.267	52,461	5.85	43%	1407
Complement C1q subcomponent subunit B	P02746	C1QB	Complement activation, innate immune system, inner ear development, proteolysis	Cytosol, endoplasmatic reticulum, mitochondria, peroxisome	0.095	0.132	0.047	1.38	26,704	8.83	41%	656
Glyceraldehyde-3-phosphate dehydrogenase	P04406	GAPDH	Oxidoreductase, transferase, apoptose, glycolysis, regulation of translation	Cytoplasm, cytoskeleton, membrane, nucleus	0.019	0.027	0.004	1.393	36,030	8.57	66%	1113
Apolipoprotein E	P02649	APOE	Cholesterol metabolism, lipid metabolism, lipid transport, steroid metabolism, sterol metabolism, transport protein	Chylomicron, HDL, VLDL	0.026	0.037	0.005	1.399	36,132	5.65	74%	1912
Apolipoprotein E	P02649	APOE	Cholesterol metabolism, lipid metabolism, lipid transport, steroid metabolism, sterol metabolism, transport protein	Chylomicron, HDL, VLDL	0.085	0.121	0.037	1.419	36,132	5.65	65%	1438
Prothrombin	P00734	F2	Acute-phase protein, blood coagulation, haemostasis	Extracellular	0.024	0.036	0.04	1.498	69,992	5.64	38%	1487
Apolipoprotein E	P02649	APOE	Cholesterol metabolism, lipid metabolism, lipid transport, steroid metabolism, sterol metabolism, transport protein	Chylomicron, HDL, VLDL	0.026	0.041	0.028	1.588	36,132	5.65	44%	884
Complement C1q subcomponent subunit A	P02745	C1QA	Complement pathway, immune defence, innate immune system	Collagen, complement C1 complex, exosomes	0.068	0.143	0.011	2.109	26,000	9.26	20%	320
